# Yap and Taz regulate retinal pigment epithelial cell fate

**DOI:** 10.1242/dev.119008

**Published:** 2015-09-01

**Authors:** Joel B. Miesfeld, Gaia Gestri, Brian S. Clark, Michael A. Flinn, Richard J. Poole, Jason R. Bader, Joseph C. Besharse, Stephen W. Wilson, Brian A. Link

**Affiliations:** 1 Department of Cell Biology, Neurobiology and Anatomy, Medical College of Wisconsin, Milwaukee, WI 53226, USA; 2 Department of Cell and Developmental Biology, UCL, London WC1E 6BT, UK

**Keywords:** Eye development, Ocular morphogenesis, Zebrafish, Tfec, Hippo signaling, Sveinsson's chorioretinal atrophy, Choroid fissure, Coloboma, Directed differentiation of stem cells

## Abstract

The optic vesicle comprises a pool of bi-potential progenitor cells from which the retinal pigment epithelium (RPE) and neural retina fates segregate during ocular morphogenesis. Several transcription factors and signaling pathways have been shown to be important for RPE maintenance and differentiation, but an understanding of the initial fate specification and determination of this ocular cell type is lacking. We show that Yap/Taz-Tead activity is necessary and sufficient for optic vesicle progenitors to adopt RPE identity in zebrafish. A Tead-responsive transgene is expressed within the domain of the optic cup from which RPE arises, and Yap immunoreactivity localizes to the nuclei of prospective RPE cells. *yap* (*yap1*) mutants lack a subset of RPE cells and/or exhibit coloboma. Loss of RPE in *yap* mutants is exacerbated in combination with *taz* (*wwtr1*) mutant alleles such that, when Yap and Taz are both absent, optic vesicle progenitor cells completely lose their ability to form RPE. The mechanism of Yap-dependent RPE cell type determination is reliant on both nuclear localization of Yap and interaction with a Tead co-factor. In contrast to loss of Yap and Taz, overexpression of either protein within optic vesicle progenitors leads to ectopic pigmentation in a dosage-dependent manner. Overall, this study identifies Yap and Taz as key early regulators of RPE genesis and provides a mechanistic framework for understanding the congenital ocular defects of Sveinsson's chorioretinal atrophy and congenital retinal coloboma.

## Abstract (precis)

**Summary:** In zebrafish, the transcriptional regulators Yap/Taz and Tead are necessary and sufficient for optic vesicle progenitors to adopt retinal pigmented epithelium identity.

## INTRODUCTION

The neural retina (NR) and retinal pigment epithelium (RPE) arise from a common pool of progenitors during optic vesicle development. Specifically, in fish, cells from the outer layer of the optic vesicle migrate around its margins, as cells within the interior invaginate to form the optic cup (Picker et al., 2009; Kwan et al., 2012; Heermann et al., 2015). Those cells that remain in the outer layer of the optic cup constitute RPE progenitors, whereas the interior is made up of NR progenitors (Picker et al., 2009; Kwan et al., 2012; Heermann et al., 2015). These two retinal populations are distinguishable long before overt functional differentiation, as the inner elongated cells of the NR are morphologically distinct from the outer flattened prospective RPE cells.

Initially, optic vesicle progenitors express the same transcription factor-encoding genes (such as *lhx2*, *pax6*, *six3*, *vsx2* and *rx1/2/3*), and signaling from surrounding tissues subsequently contributes to the regionalization of the optic vesicle into prospective RPE and NR domains (eye morphogenesis is reviewed in supplementary material Movie 1; Sinn and Wittbrodt, 2013; Fuhrmann et al., 2014). Even after RPE and NR domains are established, both cell populations maintain the ability to transdifferentiate (Sinn and Wittbrodt, 2013; Fuhrmann et al., 2014). As eye development progresses, multiple signaling pathways (including BMP, FGF, Notch, WNT, SHH and TGFβ) continue to influence the expression of transcription factors that function in the differentiation and maintenance of the NR and RPE (Sinn and Wittbrodt, 2013; Fuhrmann et al., 2014). Although several transcriptional modulators (including Mitf, Otx and β-catenin) have been implicated in RPE differentiation and maintenance, none has yet been shown to mediate the initial specification of RPE cell identity. Therefore, either combinations of known transcriptional regulators or novel factors must initiate specification of the RPE. Here we provide evidence that targets of the Hippo signaling pathway are key regulators of RPE specification.

The Hippo kinase signaling cascade widely regulates apoptosis, proliferation and cell fate decisions during development by controlling the localization and stability of the transcriptional co-activators Yes-associated protein 1 (Yap1, or more commonly Yap) and WW domain containing transcription regulator 1 (Wwtr1, or more commonly Taz) (Varelas, 2014). Inactive Hippo signaling results in nuclear localized Yap and Taz and an increase in transcription of Yap/Taz target genes. The main nuclear binding partners for Yap and Taz are the Tea domain (Tead) transcription factors. There are four Tead homologs in vertebrates, which together are broadly expressed across tissues during development (Mann et al., 2007; Naye et al., 2007).

An involvement of Yap and Tead in eye development is suggested by the prominent expression of a Yap/Taz-Tead-responsive transgene in tissues and cells undergoing complex morphogenetic movements, including the eyes (Miesfeld and Link, 2014). Furthermore, heterozygous loss-of-function mutations in *YAP1* in humans can result in autosomal dominant coloboma and a mutation within the Yap-binding domain of TEAD1 causes Sveinsson's chorioretinal atrophy (SCRA), an autosomal dominant loss of RPE, choroid, and photoreceptors radiating from the optic nerve head (Fossdal et al., 2004; Williamson et al., 2014). Although these mutations and associated diseases have been described, the mechanism(s) underlying the defects is unknown.

In this study we address the roles of Hippo signaling components during zebrafish eye development. We analyzed loss-of-function mutations in both *yap* and *taz*, transgenic lines that manipulate Yap and Taz activity in a tissue-specific manner, and reporter lines that label RPE progenitors. These tools revealed roles for the Yap and Taz transcriptional co-activators in choroid fissure closure and RPE specification that are likely to be conserved between zebrafish and humans.

## RESULTS

### Yap/Taz-Tead signaling is active during optic cup morphogenesis

**Fig. 1.**
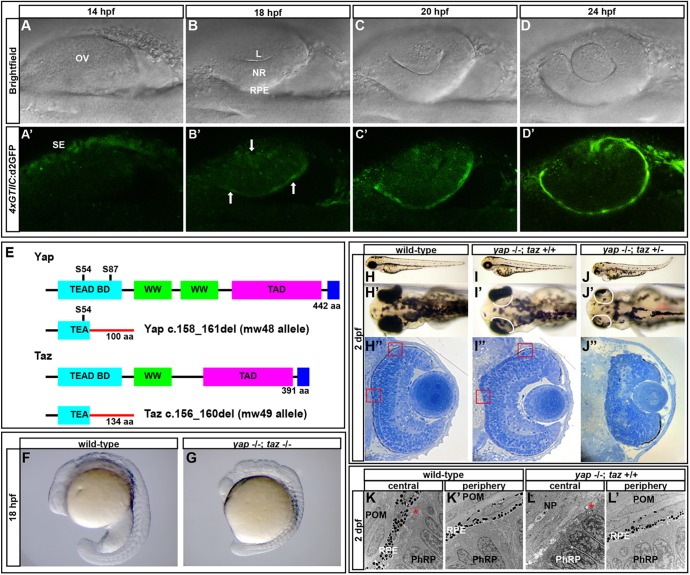
**A Yap/Taz-Tead reporter transgene is dynamically expressed during optic cup morphogenesis and *yap^−/−^* mutants exhibit RPE defects.** (A-D′) Images of live zebrafish from 14-24 hpf showing optic cup development and *4xGTIIC*:d2GFP transgene expression (green). Arrows indicate cells that are expressing the transgene while undergoing morphogenesis. (E) Schematic of wild-type and mutant Yap and Taz. Yap S54 is an essential residue for Tead binding and S87 is phosphorylated by Lats leading to cytoplasmic retention. The Yap c.158_161del and Taz c.156_160del mutants contain frameshifts resulting in early stop codons. TEAD BD, Tead transcription factor binding domain (light blue); WW, dual tryptophan motif (green); TAD, transactivation domain (fushia); PDZ domain (dark blue). (F,G) *yap^−/−^; taz^−/−^* embryos arrest by 18 hpf with multiple defects. (H-J″) Live embryos (H-J′) and sections (H″,I″,J″) of *yap^−/−^; taz^+/+^* (I-I″) and *yap^−/−^; taz^+/−^* (J-J″) showing RPE defects and additional NR defects in *yap^−/−^; taz^+/−^* mutants (J′) compared with control (H-H″). Boxed areas indicate locations of TEM analysis. (K-L′) Transmission electron microscopy analysis showing areas of normal RPE development (L′) and areas devoid of RPE (L) in *yap^−/−^* eyes. Asterisk indicates the presence of primary cilia on neuroepithelial cells*.* L, lens; OV, optic vesicle; NR, neural retina; RPE, retinal pigment epithelium; SE, surface ectoderm; POM, periocular mesenchyme; NP, neuropil; PhRP, photoreceptor progenitors.

Analysis of the *4xGTIIC*:d2GFP transgenic line (Miesfeld and Link, 2014) suggested a role for Yap/Taz-Tead activity in the developing lens, NR and RPE (Fig. 1; supplementary material Movie 2). Reporter transgene expression was evident in the ectoderm overlying the optic vesicle at 14 h post-fertilization (hpf) (Fig. 1A′) and in the lens placode and presumptive RPE by 18 hpf (Fig. 1B′). Throughout optic cup invagination transgene activity was present at low levels in NR progenitors and more prominently in RPE progenitors (Fig. 1; supplementary material Movie 2). Fluorescence is first evident in the optic cup midway through its morphogenesis, but Yap/Taz-Tead activity is likely to initiate earlier than this given the delay between transcription of the transgene and fluorescence of its protein product. To investigate the roles of Yap and Taz, we generated mutations within each gene and analyzed the consequences for eye formation.

### *yap* mutants lack RPE cells

*yap* mutant alleles were generated using transcription activator-like effector nuclease (TALEN) technology. Multiple founders containing different insertion or deletion alleles were obtained and two lines established. A 4 nt deletion, *yap c.158_161del* (*yap^mw48/mw48^*, referred to as *yap^−/−^*), resulted in a frameshift within the Tead-binding domain of Yap leading to a predicted incorrect amino acid sequence and an early stop codon (Fig. 1E). *yap^−/−^* embryos had a 3-fold decrease in *yap* mRNA (*P*=0.0009) and lacked Yap immunoreactivity (Fig. 3A-C), suggesting nonsense-mediated mRNA decay and the absence of Yap protein.

By 1 day post-fertilization (dpf), *yap^−/−^* mutants show mild heart edema, vascular hemorrhages and an impairment in RPE development (Fig. 1I-I″,L; supplementary material Fig. S1; data not shown). Some *yap^−/−^* fish survived to adulthood and none of the early phenotypes were exacerbated through the loss of maternal Yap contribution in embryos generated from *yap^−/−^* mothers. Embryos heterozygous for the *mw48* or other mutant alleles described here appeared overtly normal.

The loss of RPE in *yap^−/−^* mutants is noticeable as soon as melanization begins and becomes more apparent once retinal pigmentation is complete (Fig. 1I′,I″; supplementary material Fig. S1). RPE deficiency typically occurs at the back of the eye but can also variably occur on the lateral and ventral surfaces and can differ in phenotypic extent between eyes of the same embryo. Electron microscopy of 2 dpf *yap^−/−^* eyes revealed normal RPE cells in regions with visible pigmentation (Fig. 1L′). However, in areas lacking pigmentation there was an absence of flattened cells characteristic of either RPE or periocular mesenchyme, and NR progenitors directly abutted the forebrain neuropil (Fig. 1L). The retinal neuroepithelia appeared normal, possessed the modified primary cilia that form photoreceptor outer segments, and displayed proper retinal layering, even beneath regions lacking RPE (Fig. 1I″).

### *yap* mutants exhibit variable phenotypes including coloboma

**Fig. 2.**
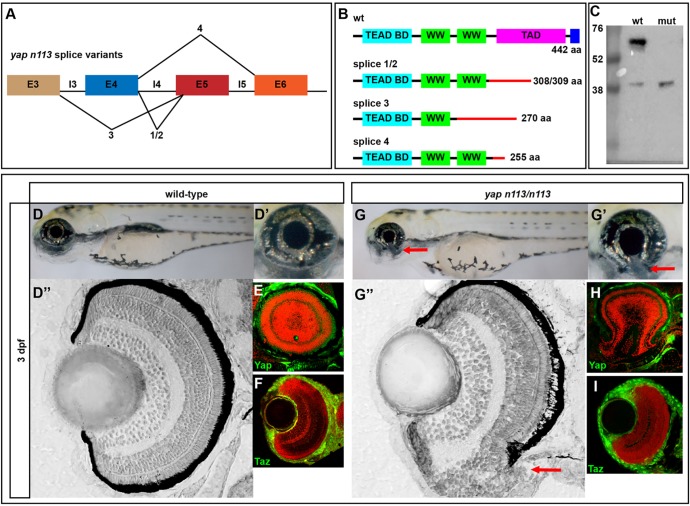
***yap^nl13/nl13^* mutants exhibit coloboma.** (A) Splice site variants elicited by the *yap^nl13^* allele. The mutation results in aberrant splicing and an early stop codon in the transactivation domain for all described products. (B) Protein schematics for wild-type and predicted mutant *yap^nl13^* allele variants. (C) Western blot showing the loss of full-length Yap in *yap^n^^l^^13/nl13^* mutants. A smaller protein product is detectable at higher levels in the mutant (∼40 kDa). (D-D″,G-G″) 3 dpf wild-type and *yap^nl13/nl13^* embryos showing coloboma (arrows) in the absence of other overt phenotypes. Plastic sections of wild-type (D″) and mutant (G″) eyes show the coloboma (arrow) phenotype and RPE deficits that are sometimes observed in the ventral retina. (E,F,H,I) Sections showing Yap and Taz proteins (green) in wild-type and *yap^n^^l^^13/nl13^* mutant eyes. Red counterstain (TO-PRO3) shows nuclei.

Although fully penetrant, the RPE phenotype in *yap^−/−^* mutants was variable and other phenotypes, including viability, showed similar variability. Additional support for phenotypic variability in *yap* mutants came from assessment of another allele, *nl13*, which exhibited a colobomatous phenotype (Fig. 2G-H) and was identified through a forward genetic screen of ENU-induced mutations. The *nl13* mutation was localized between Zv2560 and Zv8353 on chromosome 18 using bulked segregant analysis with SSLPs. *yap* lies within this interval and, given that mutations in human *YAP1* can lead to isolated and syndromic coloboma (Williamson et al., 2014), this gene was a good candidate for harboring the mutation. The genomic mutation was identified as a single base change from A to T in the splice acceptor site of intron 4. Sequencing the coding region from mutant cDNA revealed four splice variants (Fig. 2A,B), with the main isoform resulting in a deletion of 11 nt between positions 673 and 684, generating a stop codon at amino acid 309, the beginning of the transactivation domain (Fig. 2A,B). Yap immunoreactivity was still detected in *yap^nl13/nl13^* mutants (Fig. 2E,H) but western blots showed the presence of a smaller than wild-type protein (∼40 kDa versus ∼65 kDa; Fig. 2C).

To test whether the mutation in *yap* caused the coloboma phenotypes, we injected synthetic *yap-GFP* RNA into embryos from a cross between carriers and assessed phenotypic rescue. In non-injected controls, 25.45% (*n*=28/110) of embryos showed coloboma, whereas injection of 200 pg wild-type *yap* mRNA rescued the coloboma phenotype (*n*=106/110 normal; 4/110 showed coloboma). As in humans with *YAP1* mutations, the coloboma phenotype in *yap^nl13/nl13^* zebrafish embryos could be either unilateral or bilaterally symmetric. RPE deficits were observed in *yap^nl13/nl13^* embryos as in other alleles, but these were restricted to the ventral eye around the open choroid fissure. Also, as in other alleles, NR lamination was generally normal although outer retinal neurons were significantly disrupted where the coloboma was present (Fig. 2G-G″). Other than the ventral eye phenotypes, *yap^nl13/nl13^* embryos showed no overt phenotypes (Fig. 2G).

### *taz* mutant alleles enhance the *yap^−/−^* phenotype

**Fig. 3.**
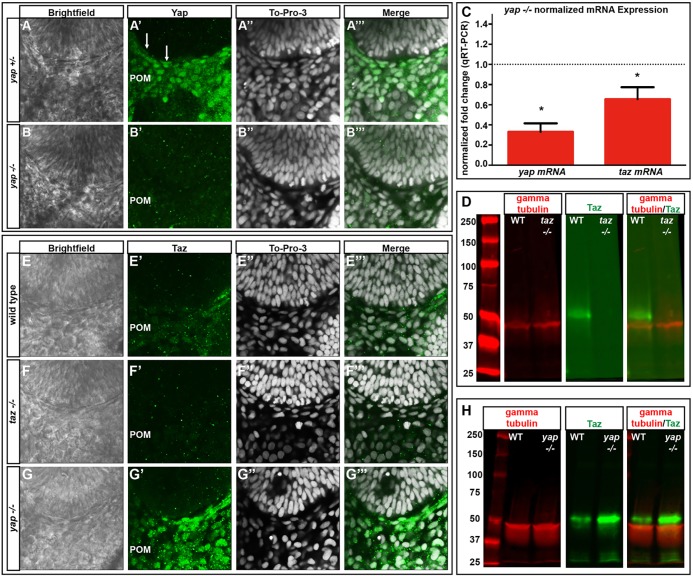
***yap* mRNA and Yap protein levels are decreased and Taz protein increased in *yap^−/−^* embryos.** (A-B‴) Yap immunoreactivity in wild-type and *yap^−/−^* eyes at 28 hpf. Yap protein is present in flattened RPE nuclei (arrows) and periocular mesenchyme (POM) in *yap^+/−^* embryos, whereas nuclear Yap staining is absent in the *yap^−/−^* mutant. (C) qRT-PCR analysis of whole embryos at 32 hpf showing a decrease in *yap* (3-fold, *P=0.0002) and *taz* (1.5-fold, *P=0.0270) mRNA in *yap^−/−^* mutants. Dotted line indicates normalized expression levels of *yap* and *taz* in wild-type embryos. An unpaired *t*-test was performed and statistical significance determined using the Holm-Sidak method. Error bars represent s.e.m. (D) Western blot showing Taz protein (∼52 kDa) in wild-type and its absence in *taz^−/−^* adult heart tissue. (E-G‴) Taz immunoreactivity in wild-type, *taz^−/−^* and *yap^−/−^* embryos at 28 hpf. (H) Western blot of Taz protein from 2 dpf wild-type (*n*=20) and *yap^−/−^* mutant (*n*=20) whole embryos.

The variable loss of RPE in *yap^−/−^* embryos (and in other alleles) can be rescued by raising embryos at 20.5°C (supplementary material Table S1). Together with the observation that some RPE develops in mutants, this suggests that another factor(s) contributes to RPE development. An obvious candidate is Taz, a homologous transcriptional co-regulator, and so we generated a mutant allele with a 5 nt deletion, *taz* *c.156_160del* (*taz^mw49^*, referred to as *taz^−/−^*). This mutation leads to a frameshift deletion in the Tead-binding domain of Taz and a resultant truncated protein containing a sequence of 82 incorrect amino acids and an early stop (Fig. 1E). Despite lacking detectable Taz protein (Fig. 3D), *taz^−/−^* embryos have no overt embryonic phenotype and survive to adulthood.

Although *taz^−/−^* RPE appears normal, the inclusion of one mutant *taz* allele within a *yap^−/−^* background (*yap^−/−^; taz^+/−^*) enhanced the loss of RPE and led to more severe body axis, heart and NR defects compared with *yap^−/−^* siblings (Fig. 1J-J″). Double-homozygous mutant (*yap^−/−^; taz^−/−^*) embryos arrest before eye morphogenesis is complete, precluding assessment of RPE development (Fig. 1F,G).

Unlike in *yap^−/−^; taz^+/+^* embryos, rearing *yap^−/−^; taz^+/−^* embryos at low temperatures did not rescue the RPE phenotype (supplementary material Table S2), suggesting that the temperature sensitivity of the *yap^−/−^* phenotype is due to redundancy with Taz (as also described in other situations; Nishioka et al., 2009)*.* Analysis of *taz* mRNA levels within *yap^−/−^* embryos did not reveal compensatory changes in transcript abundance (Fig. 3C). However, Taz immunoreactivity appeared increased at 28 hpf (Fig. 3G-G‴), higher total Taz protein levels were detected in 2 dpf *yap^−/−^* embryos (Fig. 3H) and nuclear localization appeared enhanced in *yap^nl13/nl13^* mutants (Fig. 2F,I).

### Nuclear activity of Yap/Taz-Tead is required for RPE genesis

SCRA is an autosomal dominant congenital disorder characterized by loss of RPE and photoreceptors proximal to the optic nerve head (Fossdal et al., 2004; Jonasson et al., 2007) and is caused by a tyrosine-to-histidine mutation in the Yap-binding domain of TEAD1 (Kitagawa, 2007). *yap^−/−^* embryos share phenotypic features of SCRA patients with TEAD1 deficiencies, suggesting that the consequences of Yap deficiency are mediated through Tead.

**Fig. 4.**
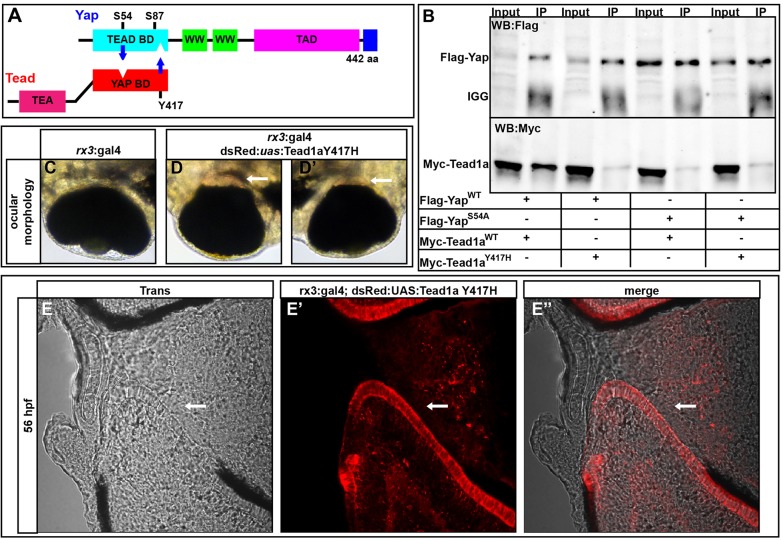
**Yap and Tead1a zebrafish protein interactions are conserved.** (A) Schematic of the zebrafish Yap and Tead binding domain (BD) interaction sites. (B) Immunoprecipitation of zebrafish Yap and Tead1a wild-type and Tead-binding-deficient Yap (Yap S54A) and Yap-binding-deficient Tead1a (Tead1a Y417H) isoforms. All the mutated protein variants lose the ability to interact, in contrast to the wild-type proteins. Immunoprecipitation (IP) was with an anti-Flag antibody. (C-E″) Whole eyes (C-D′) and sections (E-E″) showing RPE loss surrounding the optic nerve head after overexpression of Tead1a Y417H. Photoreceptors are red owing to late expression of the *rx3*:Gal4 driver in these cells. Arrows indicate areas lacking RPE.

In order to determine if a lack of Yap-Tead activity is responsible for RPE loss, we first tested whether the Yap and Tead binding domains are conserved in zebrafish. We generated plasmids encoding wild-type Tead1a, a mutant version equivalent to the YAP1 binding-deficient allele of SCRA patients (Tead1a Y417H), wild-type Yap, and a variant with a mutation within the putative Tead-binding domain (Yap S54A) (Zhao et al., 2008; Chen et al., 2010) (Fig. 4A). Consistent with studies in other species, transfection assays in HEK293 cells showed that zebrafish Yap and Tead1a are able to interact with each other, whereas the mutant variants Yap S54A and Tead1a Y417H are unable to bind (Fig. 4B). Additionally, overexpression of the autosomal dominant Tead1a Y417H allele within the optic vesicle resulted in RPE loss around the optic nerve, similar to observations in SCRA patients (Fig. 4C-E′).

**Fig. 5.**
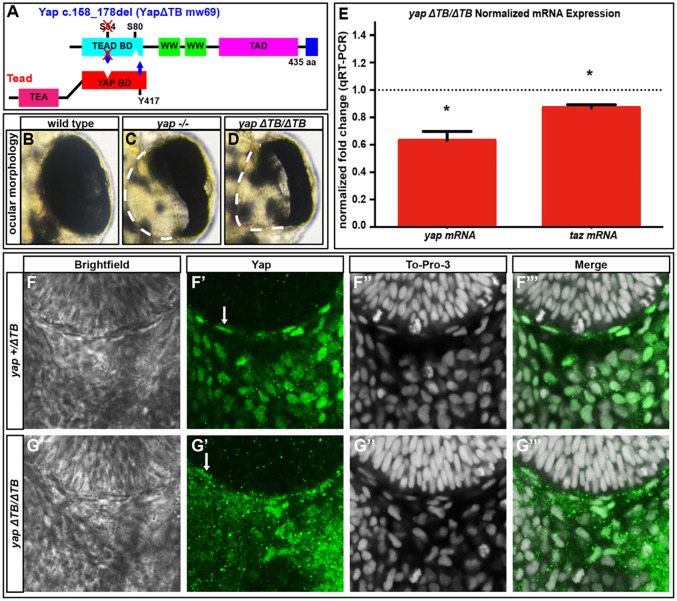
**Tead-binding-deficient *yap^ΔTB/ΔTB^* mutants lack RPE but maintain *yap* mRNA and Yap protein levels.** (A) The Tead-binding-deficient *yap^ΔTB/ΔTB^* zebrafish mutant. (B-D) 48 hpf whole eyes showing that *yap^−/−^* and *yap^ΔTB/ΔTB^* mutants lose a subset of RPE cells. Dashed lines indicate the border of the eye. (E) qRT-PCR analysis of whole embryos at 32 hpf revealing a decrease in *yap* (1.6-fold, *P=0.0052) and *taz* (1.15-fold, *P=0.0038) mRNA in *yap^ΔTB/ΔTB^* mutants. Dotted line indicates the normalized expression levels of *yap* and *taz* in wild-type embryos. An unpaired *t*-test was performed and statistical significance was determined using the Holm-Sidak method. Error bars indicate s.e.m. (F-G‴) Yap protein expression in *yap^+/ΔTB^* (F-F‴) and *yap^ΔTB/ΔTB^* (G-G‴) at 28 hpf. Yap protein is present in flattened RPE nuclei (arrows) and periocular cells in *yap^+/ΔTB^* and *yap^ΔTB/ΔTB^* embryos.

To further investigate the role of Tead in Yap-mediated RPE genesis, we generated a Tead domain-specific *yap* mutant allele. A *c.158_178del* (*yap^mw69/mw69^*, referred to as *yap^ΔTB/ΔTB^*) deletion in *yap* created a seven amino acid in-frame deletion that eliminates the critical S54 and surrounding amino acids (Chen et al., 2010) within the Tead-binding domain (Fig. 5A). *yap^ΔTB/ΔTB^* mutants had slightly lower levels of *yap* mRNA (Fig. 5E). Although Yap protein was still detected (Fig. 5F-G‴) in *yap^ΔTB/ΔTB^* embryos, loss of RPE was still evident (Fig. 5B-D).

Additional evidence for a nuclear role of Yap in RPE genesis came from analysis of the consequences of overexpression of dominant-negative forms of Yap and Taz that contain nuclear localization sequences but lack transactivation domains (NLS-YapDN, NLS-TazDN) (Cao et al., 2008; Miesfeld and Link, 2014). When either dominant-negative Yap or Taz was mosaically overexpressed in RPE/retinal precursors using *vsx2*:Gal4 (Miesfeld and Link, 2014) or *rx3*:Gal4, loss of RPE was observed (Fig. 8E-G; supplementary material Fig. S2). By contrast, expression of either dominant-negative protein in periocular mesenchymal cells using a *foxc1b*:Gal4 driver did not result in RPE defects (not shown). Collectively, these results suggest that Yap/Taz-Tead-dependent transcription is required autonomously within optic cup progenitor cells to generate RPE.

### Optic vesicle proliferation and apoptosis are unaffected in *yap^−/−^* mutants

**Fig. 6.**
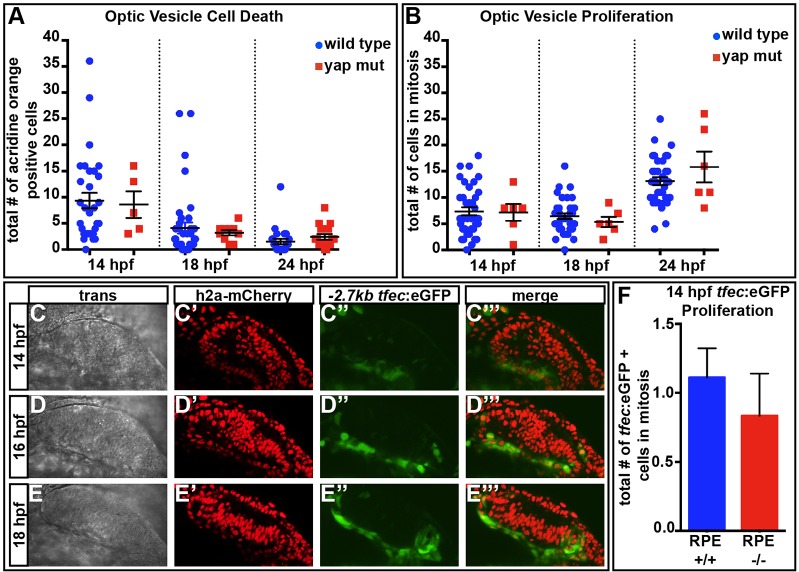
**Cell death and proliferation are normal in *yap^−/−^* eyes.** (A) The numbers of dying cells identified by Acridine Orange staining do not differ in *yap^−/−^* mutants at 14 (*P*=0.8465), 18 (*P*=0.6542) or 24 (*P*=0.2558) hpf as compared with wild type. Numbers of eyes analyzed: 14 hpf, wt *n*=31, *yap^−/−^ n*=5; 18 hpf, wt *n*=39, *yap^−/−^ n*=10; 24 hpf, wt *n*=24, *yap^−/−^ n*=16. (B) Eye field mitotic cell counts do not differ between *yap^+/^*^?^ (wt) and *yap^−/−^* at 14 (*P*=0.9205), 18 (*P*=0.4329) and 24 (*P*=0.2222) hpf. Numbers of eyes analyzed: 14 hpf, wt *n*=38, *yap^−/−^ n*=6; 18 hpf, wt *n*=38, *yap^−/−^ n*=6; 24 hpf, wt *n*=36, *yap^−/−^ n*=6. (C-E‴) Time-course of expression of *tfec*:eGFP in prospective RPE cells. (F) Mitotic cell counts of *tfec*:eGFP^+^ cells do not differ between wild-type and *yap^−/−^* eyes at 14 hpf (*P*=0.5408). Numbers of eyes analyzed: wt *n*=18, *yap^−/−^ n*=6. *P*-values were obtained using an unpaired *t*-test with equal s.d. Error bars indicate s.e.m. Wild type included full RPE coverage, whereas *yap^−/−^* showed some RPE loss.

Two well-reported functions of the Yap/Taz-Tead interaction are the control of cell proliferation and death. However, there was no obvious difference in proliferation or cell death in *yap^−/−^* eyes at 14 hpf, before markers of pigmentation are detected, at 18 hpf, the onset of observed Yap/Taz-Tead reporter transgene activity, or at 24 hpf, when RPE loss is first detected (Fig. 6A,B). To more specifically assess the proliferation of prospective RPE cells, we used a *−2.7 kb tfec*:eGFP transgene that is expressed (as is the *tfec* gene; Lister et al., 2011) in presumptive and definitive RPE cells (Fig. 6C-E‴; supplementary material Movies 3 and 4). When analyzed at 14 hpf, there was no difference in the numbers of proliferating *−2.7 kb tfec*:eGFP-positive cells between *yap^−/−^* and sibling controls (Fig. 6F).

To further test the potential significance of cell death and proliferation as contributors to the mutant phenotype, each process was inhibited during optic cup morphogenesis. To ameliorate cell death, *yap* mutants and their wild-type siblings were injected with *tp53* morpholino (Robu et al., 2007) and *bcl-xl* mRNA (Sidi et al., 2008). Although normal apoptosis in the lens epithelium was inhibited in all embryos, the *yap* mutant RPE phenotype was not rescued (not shown). Additionally, impairing proliferation in wild-type embryos from 10.5 to 26 hpf with hydroxyurea and aphidicolin did not phenocopy the RPE loss seen in *yap* mutants (not shown). These results suggest that neither changes to proliferation nor cell death are major contributing factors to RPE loss and, therefore, that Yap activity may impart RPE identity upon bi-potential optic vesicle cells.

### Yap and Taz are essential for the generation of RPE

**Fig. 7.**
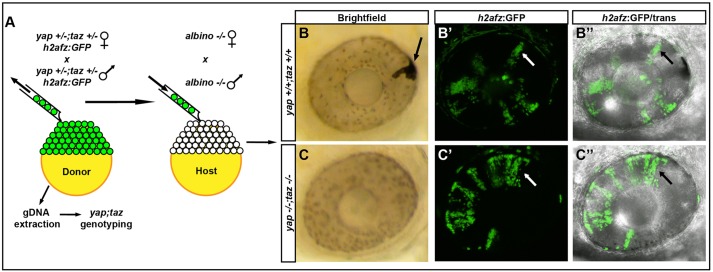
***yap^−/−^; taz^−/−^* transplanted cells do not contribute to the RPE.** (A) Methods used to analyze the contribution of transplanted cells to the RPE and NR. (B-C″) Examples of transplanted wild-type (B-B″) and *yap^−/−^; taz^−/−^* (C-C″) cells in 48 hpf eyes. The arrow in B indicates a pigmented transplanted cell forming RPE in the albino host; other black/white arrows indicate H2a-GFP^+^ clones in NR.

Our results suggest that Yap/Taz function might be essential for cells to adopt RPE identity; however, our genetic analyses did not allow us to definitively show this because *yap^−/−^; taz^−/−^* embryos arrest before RPE begins to pigment. Consequently, to test whether retinal precursors lacking Yap and Taz could form RPE, we transplanted fluorescently labeled *yap; taz* mutant cells into albino embryos that carried wild-type *yap* and *taz* alleles. Albino embryos can generate xanthophores, iridophores and leucophores but not the melanin pigment found in RPE cells (Streisinger et al., 1986; Tsetskhladze et al., 2012). Therefore, we could easily assess whether the fluorescently labeled optic vesicle cells of various *yap; taz* genotypes were able to generate RPE (Fig. 7A-C′). We scored the presence/absence of pigmented RPE in eyes that contained clones of transplanted cells in the NR to ensure that transplanted mutant cells were properly targeted to the eye field of the host blastula/gastrula.

In mosaic analyses, cells of all genotypes contributed to both NR and RPE, except for *yap^−/−^; taz^−/−^*, which only contributed to NR (Table 1). *yap^−/−^; taz^−/−^* cells were able to form neural crest-derived melanocytes indicating that the melanization pathway is functional (data not shown). These results suggest Yap and Taz are essential for optic vesicle cells to contribute to the RPE.

**Table 1.**
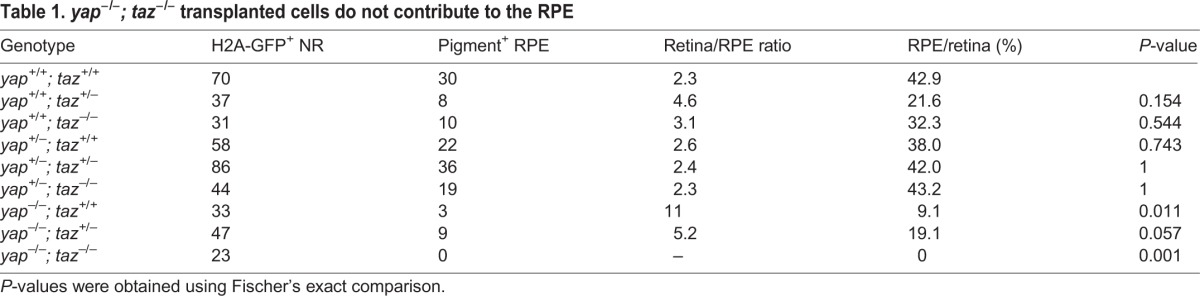
***yap^−/−^; taz^−/−^* transplanted cells do not contribute to the RPE**

**Fig. 8.**
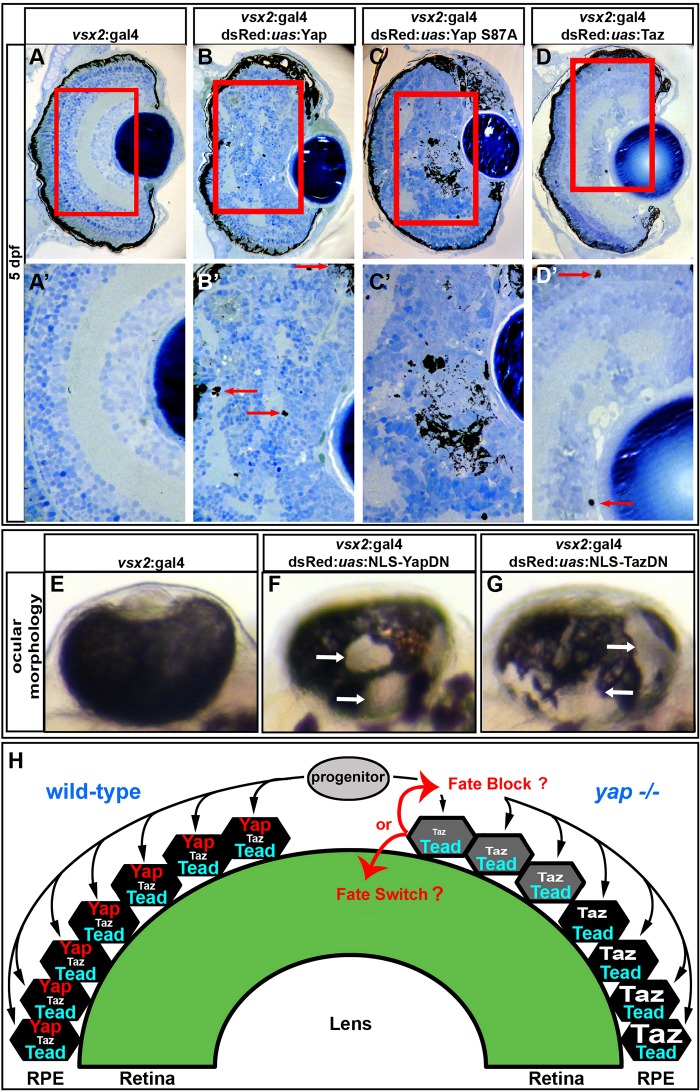
**Overexpression of gain- and loss-of-function *yap* transgenes alters RPE and NR cell fate.** (A-D′) Images showing that overexpression of wild-type Yap (B,B′), constitutively active Yap (Yap S87A) (C,C′) and Taz (D,D′) induces ectopic pigmentation and retinal disorganization at 5 dpf, as compared with the control (A,A′). Arrows indicate the presence of ectopic pigment cells. (E-G) Mosaic overexpression of dominant-negative forms of Yap (F) and Taz (G) results in ectopic loss of RPE cells at 48 hpf, as compared with the control (E). Arrows indicate areas devoid of RPE cells. (H) Model of zebrafish RPE development. Bipotent progenitor cells assume either an RPE or NR fate based on Yap/Taz activity. In the absence of *yap* (right hemisphere of the eye cup), progenitors contributing to the central retina cannot form RPE. Those progenitors that contribute to the peripheral eye cup can upregulate Taz during their more lengthy migration and therefore form RPE.

Complementing the loss-of-function analyses, *vsx2*:Gal4-driven overexpression of wild-type Yap, constitutively active Yap (Yap S87A) and, to a lesser extent, wild-type Taz, in retinal progenitors led to ectopic pigmentation as well as disrupted lamination within the NR at 5 dpf (Fig. 8A-D′). To determine if the morphological changes were reflected in altered retinal mRNA expression profiles we sequenced RNA from 36 hpf eyes overexpressing Yap S87A, which generated the strongest ectopic RPE phenotype. The top 20 transcripts upregulated by Yap S87A included *cyr61* and *ctgf*, which are established targets of Yap-Tead signaling, and both were verified as upregulated by RT-qPCR (supplementary material Table S3, Fig. S3). Other upregulated mRNA transcripts included many implicated in RPE and pigment cell genesis, differentiation and function (Table 2). Upregulation of the RPE-specific *dct* gene was verified by *in situ* hybridization in 48 hpf retinae (supplementary material Fig. S4A-B″). Additionally, destabilized GFP expressed from a *ctgf* promoter transgene (*−1.0 kb ctgfa*:d2GFP) showed increased fluorescence in RPE cells and ectopic expression in the NR (supplementary material Fig. S3B-F″) in response to *vsx2*-mediated Yap overexpression.

**Table 2.**
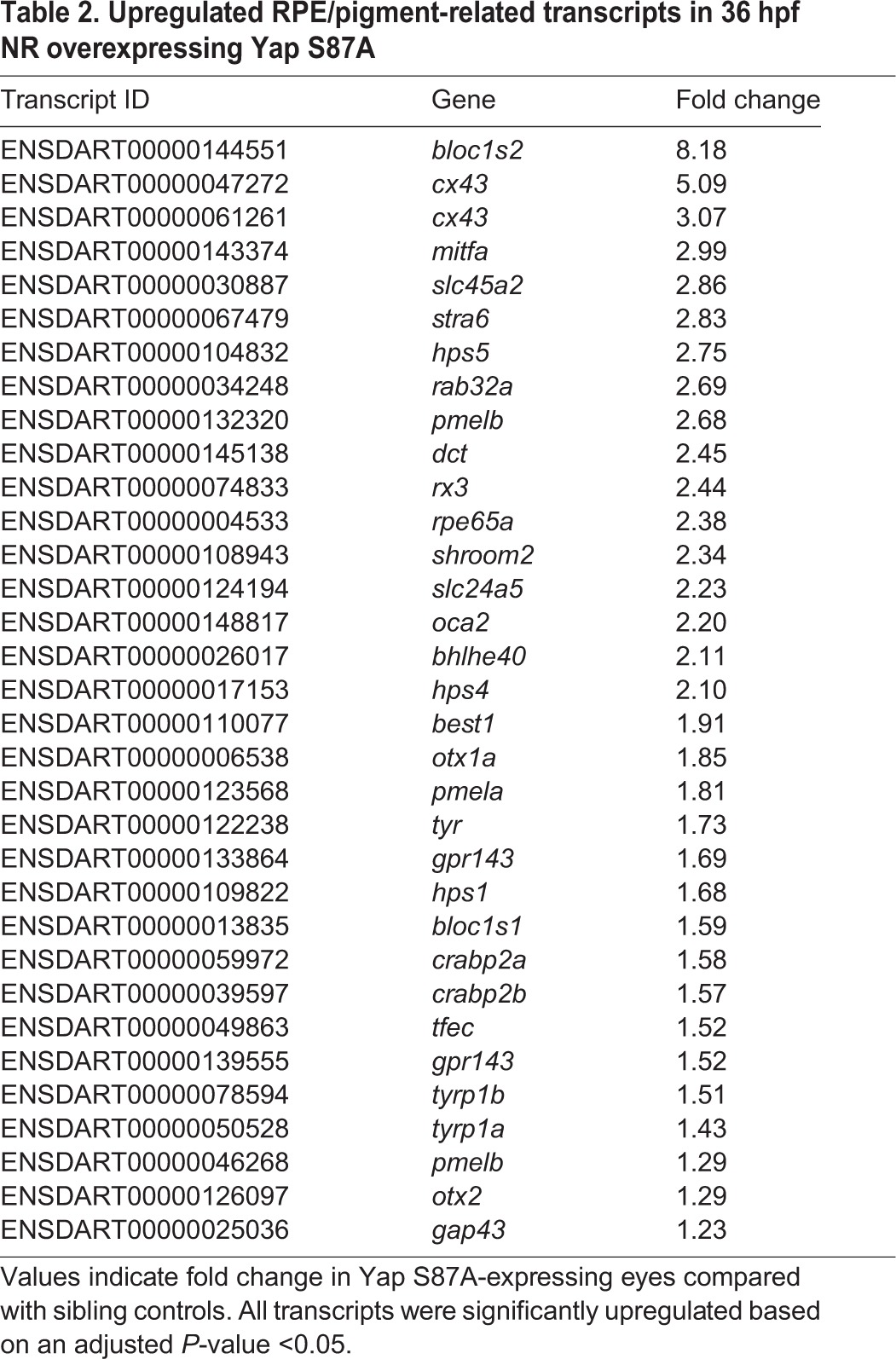
**Upregulated RPE/pigment-related transcripts in 36 hpf NR overexpressing Yap S87A**

The *vsx2*:Gal4 transgene is mosaically expressed in RPE progenitor cells as well as NR progenitors (Miesfeld and Link, 2014) and thus if exogenous Yap were to trigger the migration of existing RPE cells into the NR, this could contribute to the phenotype. However, RPE-specific *−2.7 kb tfec*:Gal4-driven overexpression of Yap S87A did not lead to any obvious migration of Yap S87A-positive RPE cells into the NR (supplementary material Movies 5 and 6, Fig. S4C-C‴). Altogether, these results support the idea that Yap and Taz are necessary and sufficient to promote optic cup cells to adopt RPE cell fate.

## DISCUSSION

Our study suggests a model for RPE genesis in which strong Yap-Tead transcriptional activity within bipotential optic cup progenitors drives RPE fate. Strong expression of the Yap/Taz-Tead-responsive *4xGTIIC*:FP transgene in presumptive RPE suggested a potential role for the downstream transcriptional components of the Hippo signaling pathway in RPE development. Although the *4xGTIIC*:FP transgene reports both Yap and Taz activity, Yap appears to be the main regulator of RPE cell fate. Consistent with this idea, *yap^−/−^* embryos are devoid of a subset of RPE cells, whereas *taz^−/−^* embryos develop normal RPE. The difference in phenotypes is consistent with the low levels of Taz expression in optic vesicle cells. Although *taz^−/−^* embryos develop normal RPE cells, compromising Taz function on a *yap^−/−^* background enhanced the loss of RPE cell phenotype as compared with *yap^−/−^* mutants alone. These observations place Yap and Taz as key regulators of RPE fate determination.

An interesting facet of the RPE phenotype in *yap^−/−^* embryos is the incomplete loss of RPE and the ability to rescue the phenotype through low-temperature rearing, implying that another protein, or proteins, can supplement for the loss of Yap. Based on our observations, we believe Taz is responsible for this compensation. Although the levels of *taz* transcript in *yap^−/−^* embryos are not increased, the *yap^−/−^* RPE phenotype is *taz* gene dosage-dependent and Taz protein was elevated in *yap^−/−^* mutants. That *taz* mRNA levels are unaffected is consistent with observations in *yap^−/−^* cardiomyocytes, which also show *taz* gene dosage-dependent phenotypes (Xin et al., 2013). The decreased *taz* mRNA levels could be the result of increased nuclear localization of Taz, which negatively feeds back on *taz* transcription*.* Changes in the stability of Taz might contribute to the increased Taz protein levels accompanying decreased *taz* mRNA. Taz contains a phosphodegron site that is primed by Lats phosphorylation, resulting in subsequent ubiquitylation and degradation (Liu et al., 2010). Potentially, the stability of Taz could be changed by a decrease in phosphorylation of the phosphodegron in *yap* mutants.

The following scenario may explain the partial loss of the RPE phenotype of *yap* mutants. Within *yap^−/−^* embryos Taz stability might be enhanced, but it might take time for translated Taz protein to reach sufficient levels for RPE specification. If so, those progenitor cells that spread and migrate further to the periphery prior to differentiation may have time to stabilize Taz and consequently generate RPE (Fig. 8H). The progenitor cells in the central eye differentiate at or near their site of origin and, consequently, Taz may have insufficient time to reach the levels necessary to promote RPE fate (Fig. 8H). Because RPE and NR differentiate from the same pool of cells within the optic cup, the cells that fail to adopt RPE fate in *yap^−/−^* mutants might instead become NR progenitors, but it is also possible that these cells assume extra-ocular cell type fates (Fig. 8H).

Slowing development by low-temperature rearing might allow sufficient Taz levels to accumulate, enabling compensatory Taz activity within central progenitor cells, thus explaining the rescue of *yap^−/−^* phenotypes by temperature shift. Consistent with this idea, low-temperature rearing does not rescue RPE when Taz is also abrogated. Importantly, cell transplantations indicated that *yap^−/−^; taz^−/−^* double-homozygous mutant cells are completely incapable of generating RPE. Similarly, dominant-negative Yap or Taz disrupts RPE genesis whereas, conversely, overexpression of either Yap or Taz can drive RPE fate from optic cup progenitor cells.

We do not know the upstream mechanism controlling Yap/Taz activity during RPE genesis, although it is likely that cross-talk with other signaling pathways is involved. WNT, BMP and TGFβ signaling all control aspects of RPE/NR development through the control of Smads and β-catenin (Sinn and Wittbrodt, 2013; Fuhrmann et al., 2014), both of which have been shown to interact with Yap or Taz (Varelas et al., 2010; Azzolin et al., 2012). Consequently, it is possible Yap/Taz-Tead activity regulates the transcription of RPE-specific genes in concert with the transcription factor targets of these pathways.

Yap and Taz have multiple binding partners within the cytoplasm and nucleus, the best-characterized of which are the Tead transcription factors. Our data imply that Yap/Taz-Tead interactions are responsible for RPE genesis. First, the *4xGTIIC*:FP Tead-responsive transgenic reporter is active early within RPE progenitor cells. Second, nuclear localized dominant-negative Yap and Taz proteins can cause RPE loss. Finally, loss of RPE was observed with overexpression of the Yap-binding-deficient Tead1a Y417H form, and within eyes in the *yap* Tead-binding-mutant allele. Despite the loss of RPE cells within *yap^ΔTB/ΔTB^* mutants, the embryos maintained wild-type levels of *yap* mRNA and robust levels of Yap protein. Loss of Tead binding by Yap in other contexts also results in similar phenotypes to *yap* loss-of-function and Yap-binding-deficient alleles of *TEAD1* (Fossdal et al., 2004; Williamson et al., 2014). SCRA patients carry an autosomal dominant mutation in the Yap-binding domain of TEAD1 and the loss of central RPE in these patients mimics the phenotypes observed in zebrafish *yap^−/−^* and Tead1a Y417H overexpression embryos (Fossdal et al., 2004; Jonasson et al., 2007). Given these similarities, our data suggest that the congenital defects in the RPE of SCRA patients arise from autonomous Yap/Tead functional deficits of cells within the optic cup, as in *yap* loss-of-function zebrafish mutants.

As observed in cases of human mutations of *YAP1* we see phenotypic variability within and across our different zebrafish *yap* mutant genotypes. Notable differences occur when *yap^−/−^* phenotypes are compared with *yap^nl13/nl13^* phenotypes. The loss of RPE is less evident in *yap^nl13/nl13^* mutants but there is a striking coloboma, similar to that observed in human patients with *YAP1* mutations (Williamson et al., 2014). We have not resolved why coloboma is evident in this mutant, but one intriguing possibility is that it could be a consequence of compromised RPE generation in the ventral retina. This might introduce mechanical disruptions to the movements of cells lining the fissure that do not occur when RPE generation is disrupted in the central retina. In this regard, it is interesting to note that recent studies of *yap* mutant medaka fish suggest that this pathway is a crucial regulator of actomyosin contractility (Porazinski et al., 2015). To address this issue will require manipulation of Yap/Taz-Tead activity in different populations of cells of the ventral retina to determine the cause of the colobomatous phenotype.

Our data reveal a role for Yap and Taz activity in regulating the ability of optic vesicle cells to adopt RPE cell fate. The idea that combined Yap/Taz activity is crucial for RPE fate determination is consistent with recently published reports of Yki and Yap/Taz influencing cell fate decisions in various other tissues (Nishioka et al., 2009; Zhang et al., 2011; Judson et al., 2013; Yimlamai et al., 2014). Our data potentially have implications for the efficient directed differentiation of stem cells to RPE fate – an important goal, given the initial success of stem cell-differentiated RPE cell transplants into patients with macular degeneration (Schwartz et al., 2015).

To conclude, in this study we revealed that Yap and Taz influence RPE cell fate in a gene dosage-dependent manner. The underlying mechanism is through a cell-autonomous nuclear interaction of Yap/Taz with Tead transcription factors. The elucidation of this basic mechanism offers a framework for continued investigation into the upstream and downstream protein-protein and protein-DNA interactions in eye development. The mutant lines and transgenic resources generated in this study will also facilitate resolution of the mechanistic basis of ocular phenotypes seen in patients with SCRA and congenital retinal coloboma caused by aberrant Yap/Taz-Tead activity.

## MATERIALS AND METHODS

### Mutant generation

TALENs for Yap and Taz were generated by Cellectis Bioresearch and supplied as plasmids (pTAL.CMV-T7.013535, pTAL.CMV-T7.013558, pTAL.CMV-T7.013534 and pTAL.CMV-T7.01355). The left *yap* TALEN arm targeted exon 1 (5′-TGAGGTTGAGAAAGCTG-3′) and 15 nt downstream in exon 1 was the right *yap* TALEN arm target sequence (5′-TTTGGCTCTGGCGGCGT-3′). The left *taz* TALEN arm targeted exon 1 (5′-TGCCGCAGTCTTTCTTC-3′) and 15 nt downstream in exon 1 was the right *taz* TALEN arm target sequence (5′-TGCCGGGAGTGGGAGCC-3′). mRNA was generated from the supplied plasmids using the mMessage mMachine and Poly(A) Tailing Kit (Ambion) and injected into 1- to 4-cell stage embryos at 150 pg per TALEN arm. Offspring from the *yap* or *taz* TALEN-injected embryos were screened using restriction sites within each spacer region. Once founders were identified, offspring were raised and tested for allele heterozygosity. The *nl13* allele was identified in a forward genetic screen of fish carrying N-ethyl-N-nitrosourea-induced mutations carried out by Alex Nechiporuk's group (Oregon Health and Science University). Mapping and identification of the causative mutation followed standard procedures (Valdivia et al., 2011).

### Genotyping

The Puregene Core Kit A (Qiagen) was used to extract genomic DNA (gDNA) from zebrafish tissue. Each gene region of interest was PCR amplified (supplementary material Table S4) and assessed for the presence or absence of a restriction site. The *yap* 4 bp deletion mutant (*mw48*) was detected by the addition of a *Tfi*I restriction site, while the *yap* 21 bp (*mw69*) and *taz* 5 bp (*mw49*) deletion mutants were detected by the loss of *Hin*fI.

### Generation of plasmids and transgenic lines

All plasmids were generated using Gateway (Invitrogen) entry clones and recombination in conjunction with the Tol2 Kit (Kwan et al., 2007). The 2.7 kb proximal upstream region of *tfec* was obtained through PCR of zebrafish gDNA and using recombination eGFP was inserted downstream of the *−2.7 kb tfec* promoter (*−2.7 kb tfec*:eGFP). RT-PCR was performed to generate Flag- or Myc-tagged full-length cDNA sequences for zebrafish *yap* and *tead1a.* A Tead-binding-deficient Yap (Yap S54A) was generated using QuikChange (Stratagene) site-directed mutagenesis. The Yap-binding-deficient Tead1a (Tead1a Y417H) mutant was made by including nucleotide changes within the 3′ PCR primer used for cloning. Flag-Yap, Flag-Yap S54A, Myc-Tead1a and Myc-Tead1a Y417H were inserted downstream of the CMV/SP6 promoter using recombination. Yap, Yap S87A, Taz, Myc-Tead1a Y417H and Yap and Taz dominant-negative constructs were generated as previously described (Miesfeld and Link, 2014). Transposase mRNA was injected with the fully assembled Tol2 constructs to generate each transgenic line (Kawakami, 2005). Transgenic and mutant lines are listed in supplementary material Table S5.

### Immunofluorescence

Standard methodology was used for whole-mount immunofluorescence and imaging (Clark et al., 2011). A 1:200 dilution of Yap rabbit polyclonal (Cell Signaling, 4912) or Taz/Yap (D24E4) rabbit monoclonal (Cell Signaling, 8418) and a 1:800 dilution of Alexa 488 anti-rabbit (Invitrogen) were used for Yap and Taz detection. Nuclei were labeled with TO-PRO-3 (Molecular Probes).

### Transfection assay

HEK293 cells were grown in DMEM (Gibco) supplemented with 10% FBS and penicillin and streptomycin at 37°C in 5% CO_2_. Cells were transfected with Lipofectamine 2000 (Invitrogen) according to the manufacturer's instructions. Plasmids transfected included *pTol2:CMV/SP6:Flag-Yap^WT^:pA*, *pTol2:CMV/SP6:Flag-Yap^S54A^:pA*, *pTol2:CMV/SP6:Myc-Tead1a^WT^:pA* and *pTol2:CMV/SP6:Myc-Tead1a^Y417H^:pA*.

### Immunoprecipitations and western blotting

Cells were harvested 24 h after transfection in lysis buffer (50 mM Tris-HCl pH 8.0, 150 mM NaCl, 1% NP-40) supplemented with a protease inhibitor cocktail (Roche), lysed in liquid nitrogen, centrifuged at 10,000 rpm (11,180 ***g***) for 10 min, and the supernatants used for immunoprecipitation analysis. Immunoprecipitations were performed with rabbit monoclonal anti-Myc (Cell Signaling, 2278) or rabbit monoclonal anti-Flag (Sigma-Aldrich, F7425) antibodies and protein G-sepharose beads (GE Healthcare) overnight on a rotator at 4°C. Proteins were eluted from the beads by boiling for 5 min in SDS sample buffer. Elutes were fractionated by SDS-PAGE (7.5% Mini-Protean TGX, Bio-Rad) and transferred to Immobilon FL PVDF membranes (Millipore). Membranes were incubated for 1 h at room temperature in Odyssey blocking buffer (LI-COR Biosciences), and incubated overnight at 4°C with anti-Myc or anti-Flag primary antibodies diluted 1:1000 in Odyssey blocking buffer, then with near infrared fluorescent secondary antibodies (IRDye 680RD and IRDye 800CW, LI-COR Biosciences) for 1 h at room temperature. Proteins were detected with an Odyssey infrared imager (LI-COR Biosciences).

Equivalent numbers (*n*=20) of 2 dpf wild-type and *yap^−/−^* whole embryos were de-yolked (Link et al., 2006) and processed in lysis buffer and centrifuged as previously described. The supernatants were boiled for 5 min in SDS sample buffer and all subsequent steps were as described above. Taz was detected using a 1:1000 dilution of Taz/Yap (D24E4) rabbit monoclonal (Cell Signaling, 8418).

### *In situ* hybridization

Embryos were fixed in 4% paraformaldehyde at 48 hpf and standard methodology was followed as previously described (Thisse and Thisse, 2004).

### Proliferation analysis

Embryos were collected from incrosses of *yap^+/–^; h2afz*:GFP^+^ fish and raised in 0.003% N-phenylthiourea (PTU) diluted in Instant Ocean fish water at 28.5°C before analysis, and transferred to 100% fish water after analysis. Embryos were dechorionated at 14, 18 or 24 hpf, anesthetized in 3-amino benzoic acid ethyl ester (Tricaine, Sigma-Aldrich), and mounted in 1% low-melt agarose (Fischer Scientific) in glass-bottom Petri dishes. Both eye fields were imaged for each embryo using confocal microscopy. After imaging, embryos were freed and raised individually in 48-well plates. The genotype of each embryo was determined by the loss of RPE phenotype at 48 hpf. Mitotic cells for each image were counted using ImageJ software (NIH). A cell was counted as mitotic if a metaphase plate was clearly visible in a single or recently divided nucleus. This same analysis was performed at 14 hpf for *−2.7 kb tfec*:eGFP^+^ cells. To inhibit proliferation we treated wild-type embryos with 20 mM hydroxyurea (Sigma-Aldrich), 150 μM aphidicolin (Cayman Chemical) and 3% DMSO in fish water from 10.5-26 hpf.

### Cell death analysis

Embryos were incubated at 28.5°C for 20 min in 20 ml 5 μg/ml Acridine Orange (Sigma-Aldrich) diluted in Instant Ocean embryo media at 14, 18 or 24 hpf. After incubation, embryos were briefly washed four times in PTU, anesthetized in Tricaine and embedded in 1% low-melt agarose in glass-bottom Petri dishes and imaged by confocal microscopy. Embryos were freed and reared individually in a 48-well plate. Genotypes were assessed based on the loss of RPE phenotype. Cell death counts were performed using MetaMorph (Molecular Devices). Cell death was inhibited by co-injecting 150 μM *tp53* morpholino (Robu et al., 2007) and 100 pg *bcl-xl* mRNA (Sidi et al., 2008) into 1- to 2-cell stage embryos.

### Quantitative real-time PCR analysis

All cDNA was generated using the Superscript III First-Strand Synthesis System for RT-PCR Kit (Invitrogen) per manufacturer's instructions and all qRT-PCR was performed on a CFX96 and CFX Connect Real-Time System (Bio-Rad) using SsoAdvanced SYBR Green Supermix (Bio-Rad). Analysis of *yap* and *taz* transcripts was performed on whole embryos. mRNA was extracted from dechorionated wild-type (ZDR), *yap^−/−^* and *yap^ΔTB/ΔTB^* embryos at 32 hpf using the RNeasy Plus Mini Kit (Qiagen) per manufacturer's instructions. qRT-PCR was performed using equivalent amounts of mRNA for four biological replicates of ten pooled embryos for ZDR and *yap^−/−^* embryos, while three biological replicates of ten pooled embryos were used for *yap^ΔTB/ΔTB^* embryos. All biological replicates were run in triplicate for each transcript. The zebrafish housekeeping gene *ef1a* was used for normalization.

For verification of transcripts identified as upregulated in RNA-seq, whole eye tissue from 36 hpf embryos expressing Yap S87A or non-expressing siblings was pooled (15 embryos/30 eyes) and mRNA was extracted using TRIzol (Invitrogen). All transcripts were measured in triplicate for each of three independent biological replicates analyzed. *ef1a* was used for normalization.

### Blastulae transplantation

Donor cells were transplanted and targeted into host embryos as previously described (Kemp et al., 2009). After transplantation, donor embryos were genotyped and host embryos raised individually in 48-well plates containing fish water. Retinal clones were scored between 1 and 2 dpf based on the presence of eGFP^+^ nuclei. RPE clones were assessed at 2 dpf based on pigmentation and the characteristic hexagonal shape of RPE cells (Fig. 7). Only eyes that contained H2A-GFP^+^ NR clones were assessed for RPE clones. Fischer's exact test was used to compare the RPE/NR ratio of each mutant genotype combination with the wild-type ratio.

### RNA-seq

Yap S87A and sibling control whole eyes were dissected at 36 hpf and immediately frozen on dry ice until ∼60 pooled retinas were obtained for each genotype. RNA was purified as described (Uribe et al., 2012), except that RNA was eluted in a 50 μl final volume. RNA quality was determined using an Agilent BioAnalyzer. 50 bp single-read sequencing was performed in triplicate for each genotype using an Illumina HiSeq2000 at VANTAGE (Vanderbilt University). Sequencing results were analyzed by VANGARD (Vanderbilt University). RNA-seq reads were mapped to *D. rerio* cDNA sequences from Ensembl release 66. RNA-seq data have been deposited at GEO under accession GSE71681.

## Supplementary Material

Supplementary Material
