# Supplementary material for: Yap and Taz regulate retinal pigment epithelial cell fate
Source: Development. 2015 Sep 1;142(17):3021–32. doi: 10.1242/dev.119008 (PMC4582179; doi:10.1242/dev.119008)
Supplement: Supplementary Material [file supp_142_17_3021__index.html]

Yap and Taz regulate retinal pigment epithelial cell fate — Supplementary Material 

# Yap and Taz regulate retinal pigment epithelial cell fate

## DEV119008 Supplementary Material

- Supplementary Material
