# Supplementary material for: Yap and Taz regulate retinal pigment epithelial cell fate
Source: Development. 2015 Sep 1;142(17):3021–32. doi: 10.1242/dev.119008 (PMC4582179; doi:10.1242/dev.119008)
Supplement: Supplementary Material [file supp_dev.119008_DEV119008supp.pdf]

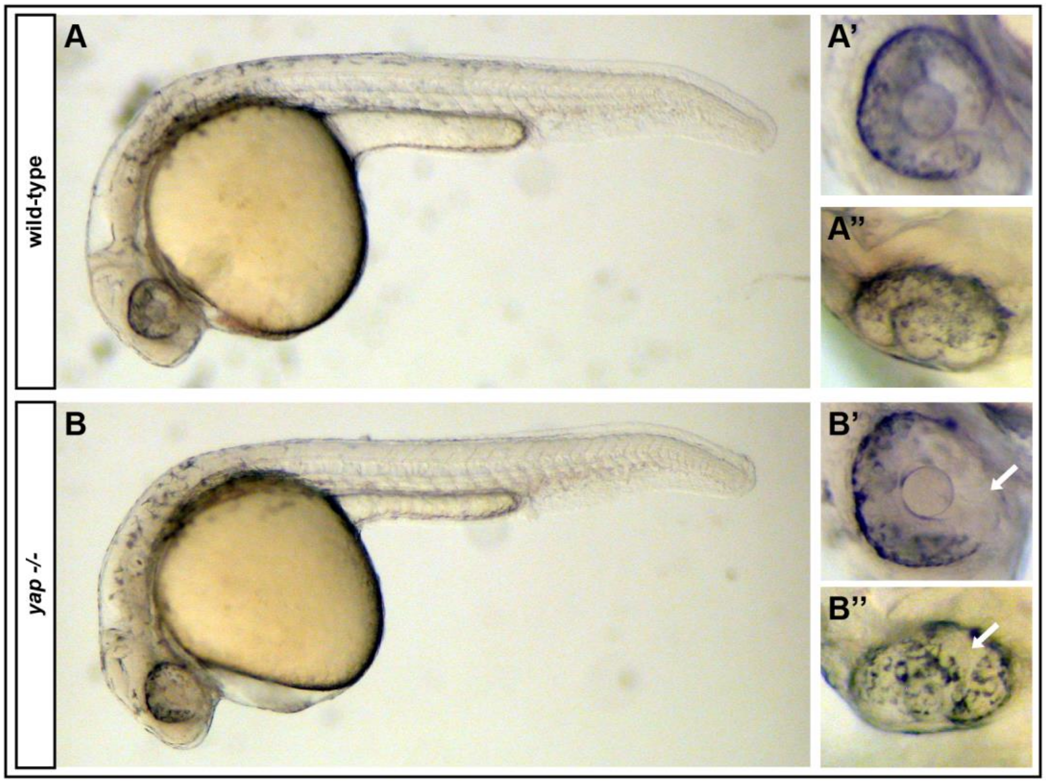

Figure S1: ***yap*$^{-/-}$ mutants exhibit RPE defects noticeable at the onset of pigmentation.**

(A-A'') A Wild-type embryo with normal RPE cells and pigmentation encompassing the whole eye globe.

(B-B'') *yap*$^{-/-}$ embryos lack RPE cells before RPE cells are completely pigmented. White arrows = areas of absent of RPE cells.

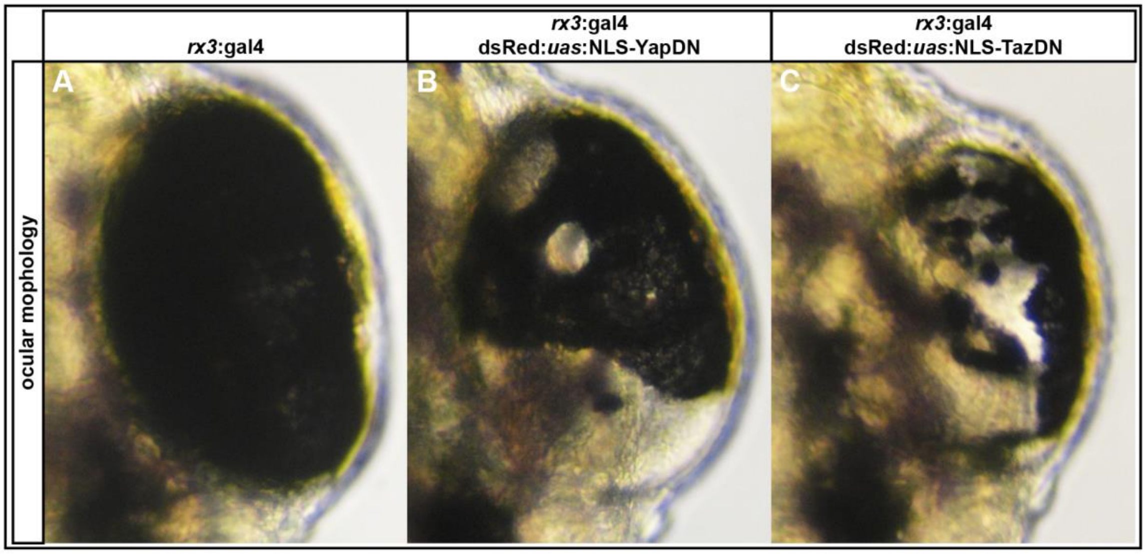

Figure S2: Overexpression of NLS-YapDN and NLS-TazDN in *rx3* positive cells results in loss of RPE.

(A-C) Examples of eyes from *rx3*:gal4+/dsRed:*uas*:NLS-YapDN and *rx3*:gal4+/dsRed:*uas*:NLS-TazDN fry lacking RPE cells at 48 hpf.

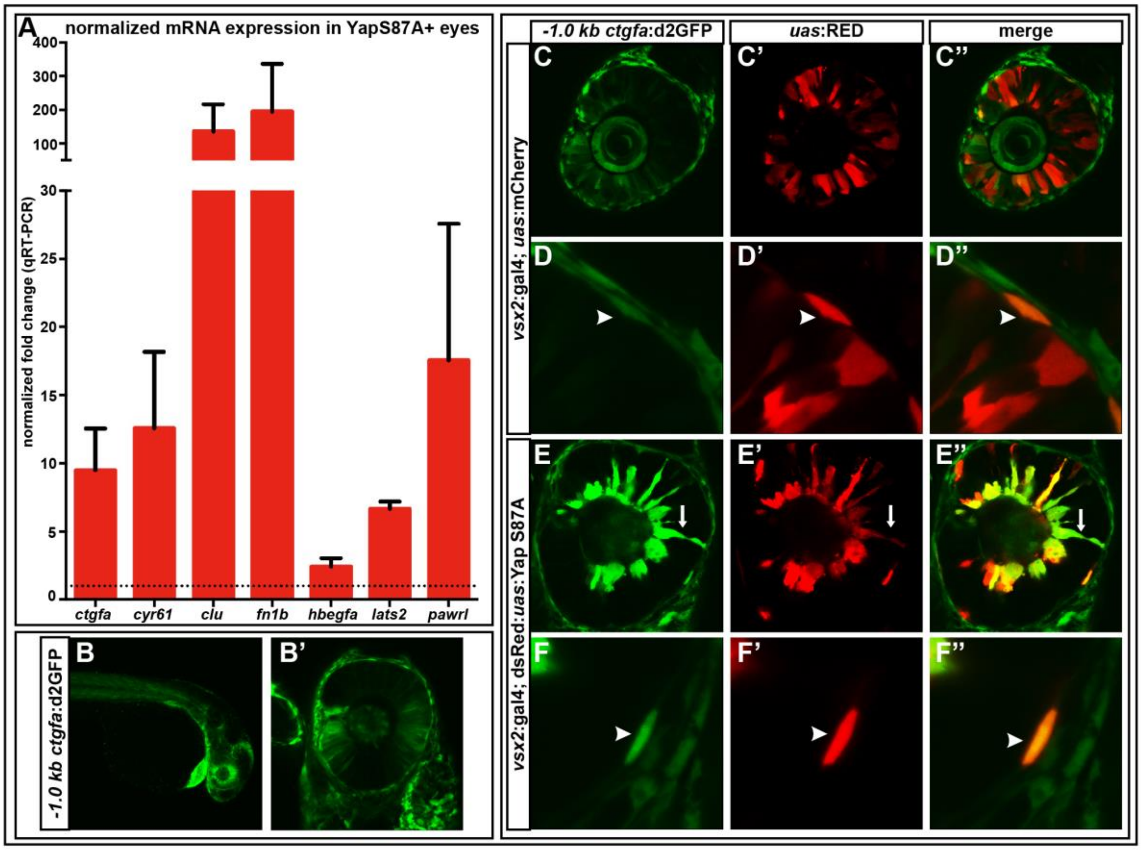

Figure S3: qRT-PCR validates mRNA transcripts that were upregulated via RNAseq in Yap S87A overexpressing 36 hpf eyes and *-1.0kb ctgfa*:d2GFP is upregulated by Yap S87A.

(A) *ctgfa* (9.5-fold, *p*=0.0508), *cyr61* (12.6-fold, *p*=0.1072), *clu* (135.5-fold, *p*=0.1705), *fn1b* (195-fold, *p*=0.2418), *hbegfa* (2.4 –fold, *p*=0.0845), *lats2* (6.7-fold, *p*=0.0005), *pawrl* (17.6-fold, *p*=0.1736). Dashed line indicates the normalized expression levels of *yap* and *taz* in wild-type embryos. An unpaired t-test was performed on mRNA expression and statistical significance was performed using the Holm-Sidak method. Error bars = s.e.m. (B-B') *-1.0kb ctgfa*:d2GFP expression is observed in the NR, RPE, heart, and other tissues at 28 hpf. (C-F'') Yap S87A overexpression results in increased *-1.0kb ctgfa*:d2GFP in RPE cells (F-F'') and ectopic NR expression (E-E'') compared to mCherry (C-D''). Arrows=ectopic NR, arrow heads=RPE expression. All embryos are 28 hpf.

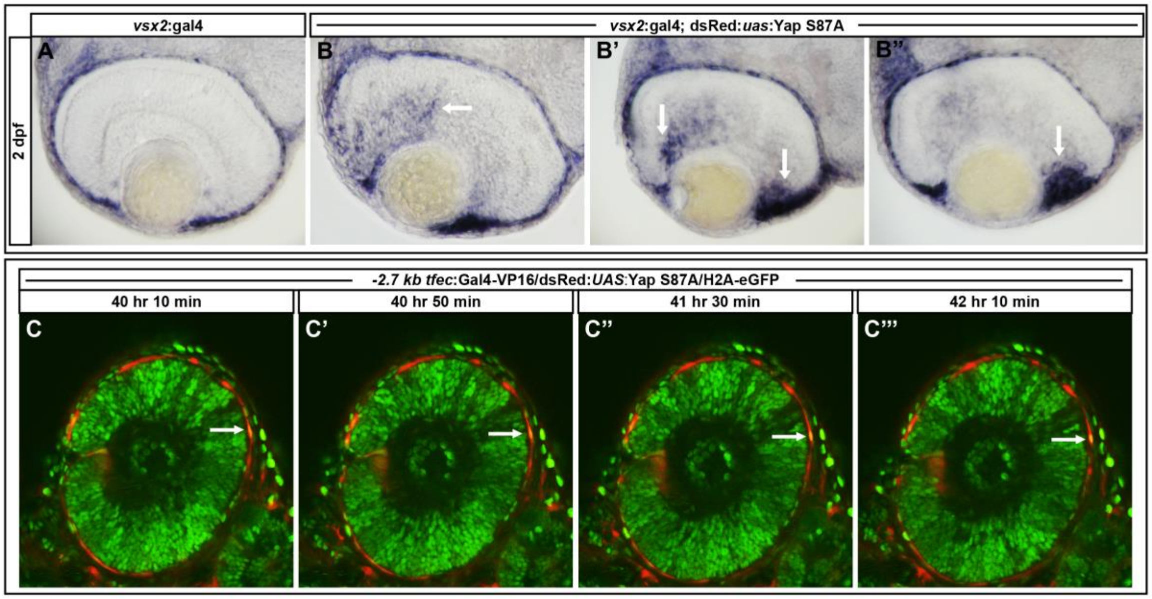

Figure S4: Overexpression of Yap S87A in *vsx2* positive cells results in ectopic expression of *dct* mRNA and -*2.7kb tfec*:Gal4-VP16/dsRed:*UAS*:Yap S87A positive cells do not migrate into the neural retina.

(A) Endogenous *dct* expression is observed in the RPE.

(B-B'') Ectopic *dct* expression is observed in the neural retina and enhanced in the ciliary marginal zone in Yap S87A overexpressing embryos. Black arrows denote ectopic *dct*.

(C-C''') RPE cells overexpressing Yap S87A do not ectopically migrate into the neural retina. Images represent a 2 hour time course from 40-42 hpf. White arrows indicate a Yap S87A positive RPE cell.

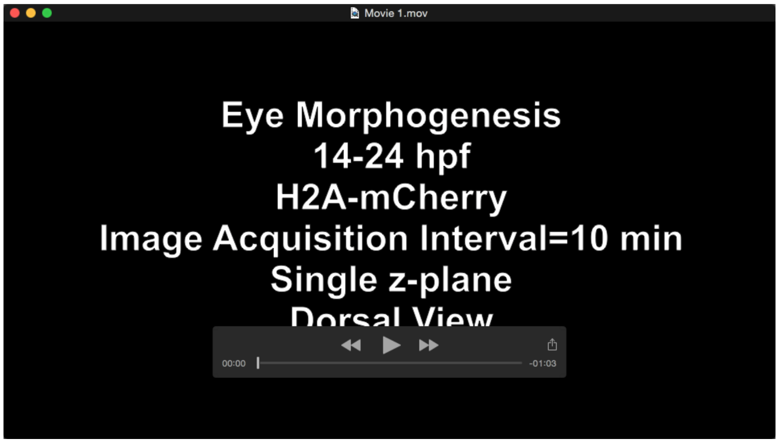

Movie 1: Time lapse of zebrafish eye morphogenesis including various transcription factors and signaling pathways involved.

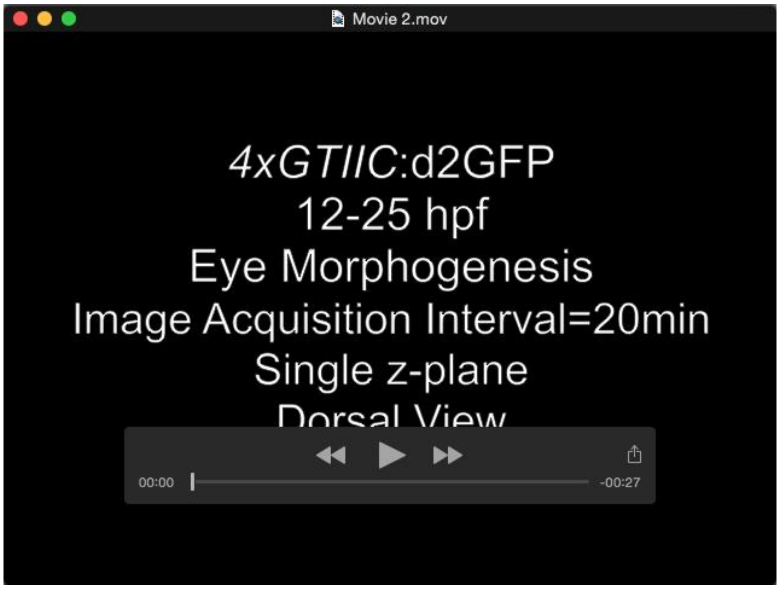

Movie 2: *4xGTIIC*:d2GFP transgene expression during optic cup morphogenesis. Arrows indicate RPE and lens expression. L=lens, NR=neural retina, RPE=retina pigment epithelium.

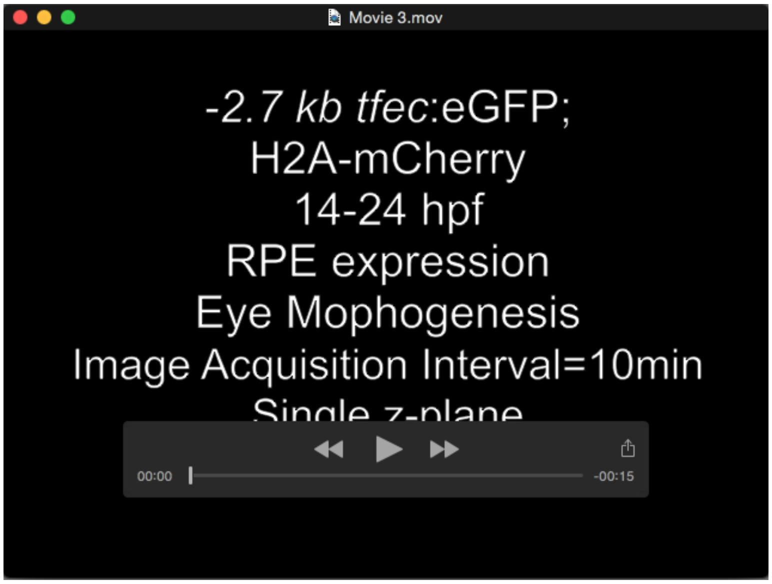

Movie 3: -*2.7kb tfec*:eGFP/*h2afz*:H2A-mCherry transgene expression from 14-24 hpf during optic cup morphogenesis.

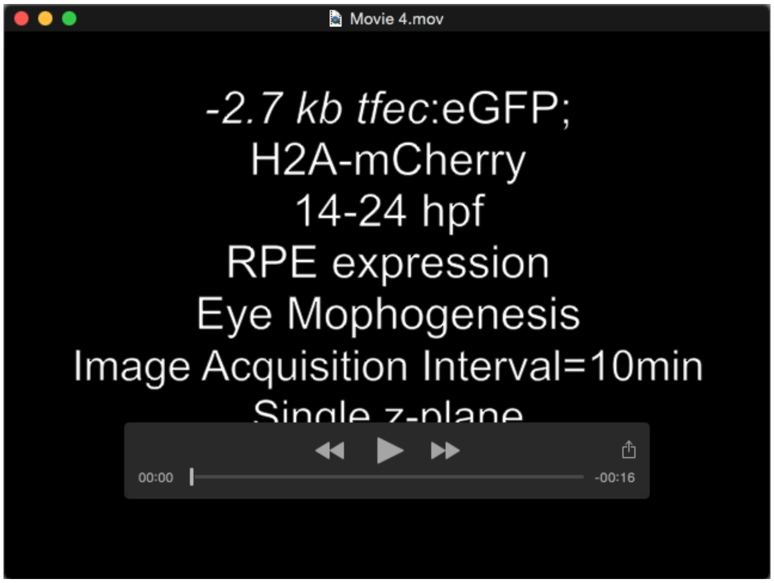

Movie 4: -*2.7kb tfec*:eGFP/*h2afz*:H2A-mCherry expression from 14-24 hpf during optic cup morphogenesis. The arrow represents a *-2.7kb tfec*:eGFP+ mitotic cell.

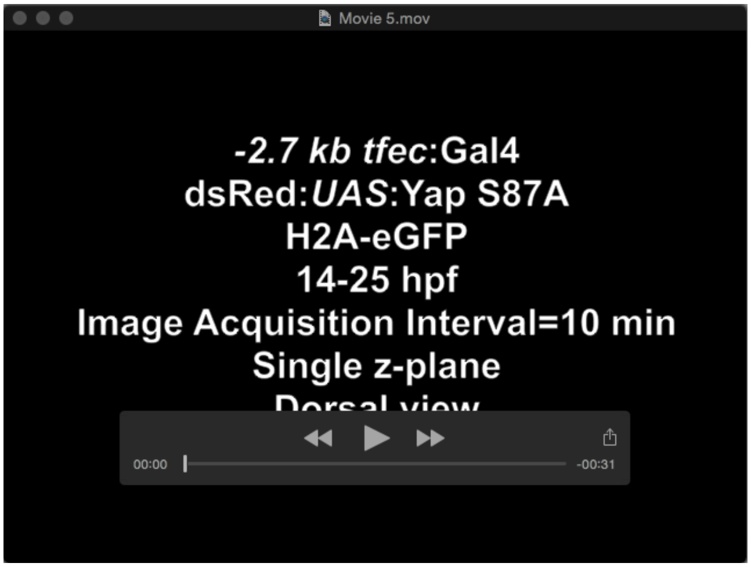

Movie 5: -*2.7kb tfec*:Gal4-VP16/dsRed:UAS:Yap S87A/H2A-eGFP expression from 14-25 hpf during optic cup morphogenesis. The arrows highlight -*2.7kb tfec*:Gal4-VP16/dsRed:UAS:Yap S87A positive cells that migrating normally around the rim of the optic cup and not into the presumptive neural retina. No abnormal RPE cell migrations were noted. The time lapse movie is played twice.

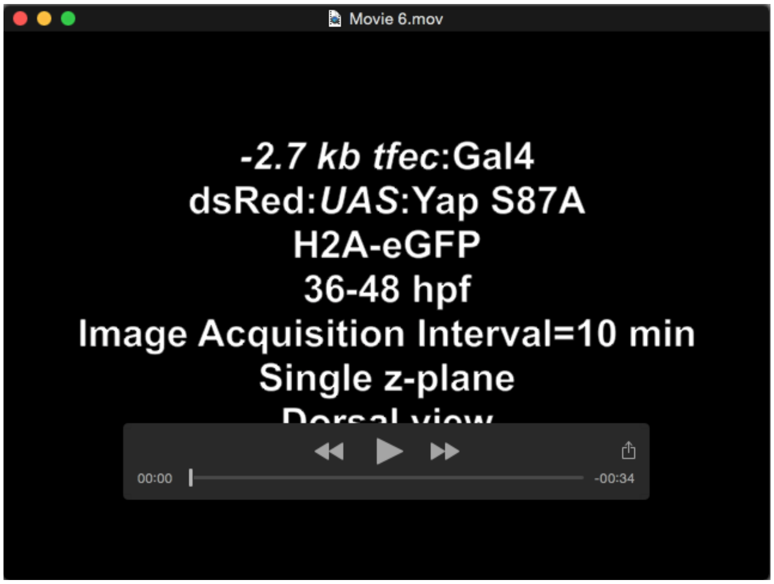

Movie 6: -*2.7kb tfec*:Gal4-VP16/dsRed:UAS:Yap S87A/H2A-eGFP expression from 36-48 hpf during retinal neurogenesis. The arrows highlight -*2.7kb tfec*:Gal4-VP16/dsRed:UAS:Yap S87A positive cells that maintain their position in the RPE and do not migrate into the neural retina. No abnormal RPE cell migration was noted. The time lapse movie is played twice.

Table S1: The loss of RPE phenotype observed in *yap*$^{-/-}$ embryos is rescued when embryos are reared at 20.5°C. Embryos were placed at 20.5°C at 70% epiboly and put back at 28.5°C at prim-10.

yap$^{+/-}$ X *yap*$^{+/-}$		
28.5°C	Predicted Values	Actual Values
Total Embryos Scored	389	389
Wild Type RPE	292	307
Mutant RPE	97	82
% of Mutant/Wild Type RPE	25.00%	21%
20.5°C	Predicted Values	Actual Values
Total Embryos Scored	389	389
Wild Type RPE	292	389
Mutant RPE	97	0
% of Mutant/Wild Type RPE	25%	0%

Table S2: The loss of RPE phenotype cannot be completely rescued by 20.5°C rearing when a mutant *taz* allele is present in the *yap*$^{-/-}$ background. Embryos were placed at 20.5°C at 70% epiboly and put back at 28.5°C at prim-10.

yap$^{+/-}$;*taz*$^{+/-}$ X *yap*$^{-/-}$;*taz*$^{+/+}$		
28.5°C	Predicted Values	Actual Values
Total Embryos Scored	136	136
Wild Type RPE	68	74
Mutant RPE	68	62
% of Mutant/Wild Type RPE	50%	46%
20.5°C	Predicted Values	Actual Values
Total Embryos Scored	135	135
Wild Type RPE	68	110
Mutant RPE	67	25
% of Mutant/Wild Type RPE	50%	19%

Table S3: The top 20 most upregulated transcripts in Yap S87A overexpressing 36 hpf eyes. Values represent the fold change of Yap S87A expressing eyes compared to sibling controls. All transcripts were significantly upregulated based on an adjusted *p*-value < 0.05.

Transcript ID	Gene	Fold Change
ENSDART00000060765	*BX323876.3*	187.12
ENSDART00000141193	*clu*	179.31
ENSDART00000109972	*BX248501.1*	91.70
ENSDART00000129496	*cyr61*	33.07
ENSDART00000018117	*ppp1r14aa*	20.69
ENSDART00000037904	*socs3b*	19.77
ENSDART00000109138	*hbegfa*	19.59
ENSDART00000104965	*plcxd1 (3 of 5)*	18.04
ENSDART00000003505	*adm (1 of 2)*	17.12
ENSDART00000112226	*apcdd1l*	16.35
ENSDART00000077951	*pcolce2b*	15.92
ENSDART00000017312	*fn1b*	15.27
ENSDART00000097460	*hmgcra*	15.23
ENSDART00000148845	*DKEY-6N3.3*	12.97
ENSDART00000063028	*ctgf*	12.50
ENSDART00000112243	*crlf1a*	11.36
ENSDART00000124465	*junbl*	10.79
ENSDART00000150088	*DKEY-119G10.4*	10.53
ENSDART00000106488	*plod2*	10.18
ENSDART00000145103	*cntfr*	9.91

Table S4: List of primers used for genotyping and qRT-PCR. E=Efficiency % of qRT-PCR primers.

Primer Name	Primer Sequence	Assay
yap 4bp/21bp F	5′-AGTCATGGATCCGAACCAGCACAA-3′	genotyping
yap 4bp/21bp R	5′-TGCAATCGGCCTTTATTTTCCTGC-3′	genotyping
taz 5bp F	5′-CTCGGCTGAAACTACTTAAGGACG-3′	genotyping
taz 5bp R	5′-CTAAACAGTGTGCAGGAATGTCC-3′	genotyping
yap qPCR F	5′-CCAGACAAGCCAGTACAGAT-3′	RT-qPCR E=95%, r^2=.997
yap qPCR R	5′-GAAGTATCTCTGTCCCGAAGG-3′	RT-qPCR E=95%, r^2=.997
taz qPCR F	5′-GCATCCAGATGGAGAGAGAG-3′	RT-qPCR E=105%, r^2=.991
taz qPCR R	5′-GCTGTTATTGGGCATGTTTC-3′	RT-qPCR E=105%, r^2=.991
ef1α exon1-2 F	5′-TCTCTCAATCTTGAAACTTATCAATCA-3′	RT-qPCR E=102.7%, r^2=.992
ef1α exon3 R	5′-AACACCCAGGCGTACTTGAA-3′	RT-qPCR E=102.7%, r^2=.992
ctgfa F	5′-CTGCACAGCCAGAGATG-3′	RT-qPCR E=120%, r^2=.990
ctgfa R	5′-CACTTCCCAGGCACTTT-3′	RT-qPCR E=120%, r^2=.990
cyr61 F	5′-CCGTGTCCACATGTACATGGG-3′	RT-qPCR E=107.7%, r^2=.987
cyr61 R	5′-GGTGCATGAAAGAAGCTCGTC-3′	RT-qPCR E=107.7%, r^2=.987
clu F	5′-GTCGCAAGTTGGTGAGAAATACC-3′	RT-qPCR E=98.7%, r^2=.900
clu R	5′-CTCCTTCATCTCCTGAGCCATC-3′	RT-qPCR E=98.7%, r^2=.900
fn1b F	5′-CAGTACTGTACAGTCAGGGGAAGC-3′	RT-qPCR E=94.7%, r^2=.970
fn1b R	5′-CACGACCGTTGTCATTACAGCC-3′	RT-qPCR E=94.7%, r^2=.970
hbegfa F	5′-CAAGCAAGGTGCATATAATGTGG-3′	RT-qPCR E=105.6%, r^2=.980
hbegfa R	5′-CTGCCAAACAAACACGGTCAC-3′	RT-qPCR E=105.6%,

		r^2=.980
lats2 F	5′-CTCCGAGAGATCCGCAAGTC-3′	RT-qPCR<br>E=93%, r^2=.974
lats2 R	5′-CACGTACAATCTGTTCAGTGTG-3′	RT-qPCR<br>E=93%, r^2=.974
pawrl F	5′-GAACAAGACCTTGCTGAAAGTG-3′	RT-qPCR<br>E=104%, r^2=.906
pawrl R	5′-CACTTCCACAATCCAAAGCGTCC-3′	RT-qPCR<br>E=104%, r^2=.906

Table S5. Transgenic and mutant zebrafish lines

Line	Reference
Tg(*4xGTIIC*:d2GFP)^{mw50}	Miesfeld and Link, 2014
Tg(dsRed.T4:*14xUAS*:Yap)^{mw63}	Miesfeld and Link, 2014
Tg(dsRed.T4:*14xUAS*:Wwtr1)^{mw64}	Miesfeld and Link, 2014
Tg(dsRed.T4:*14xUAS*:YapCA(S87A))^{mw65}	Miesfeld and Link, 2014
Tg(dsRed.T4:*14xUAS*:NLS-Yap^{DN})^{mw66}	This study
Tg(dsRed.T4:*14xUAS*:NLS-Taz^{DN})^{mw67}	This study
Tg(*–2.7 kb tfec*:eGFP)^{mw68}	This study
Tg(dsRed.T4:*14xUAS*:Myc-TeadY417H)^{mw70}	This study
Tg(*–2.7 kb tfec*:Gal4-VP16) ^{mw71}	This study
Tg(*–5.0 kb foxC1b*:Gal4-VP16)^{mw72}	This study
Tg (*–1.0 kb ctgfa*:d2GFP)^{mw75}	This study
Tg(*vsx2*:Gal4-VP16)^{mw39}	Clark et al., 2011
yap c.158_161del^{mw48}	This study
yap c.158_178del^{mw69}	This study
*wwtr1 c.156*_160del^{mw49}	*taz* mutants (this study)
yap^{nl13}	This study
Tg(*h2afv*:h2afv-GFP)^{kca6}	Pauls et al., 2001
Tg(*Ola.Rx3*:Gal4-VP16)^{vu271}	Weiss et al., 2012
albino^{b4}, *slc45a2*$^{b4/b4}$	Streisinger et al., 1986, Tsetskhladze et al., 2012

Supplementary references

Pauls, S., Geldmacher-Voss, B. and Campos-Ortega, J. A. (2001). A zebrafish histone variant H2A.F/Z and a transgenic H2A.F/Z:GFP fusion protein for in vivo studies of embryonic development. *Dev. Genes Evol.* **211**, 603-610.

Weiss, O., Kaufman, R., Michaeli, N. and Inbal, A. (2012). Abnormal vasculature interferes with optic fissure closure in lmo2 mutant zebrafish embryos. *Dev. Biol.* **369**, 191-198.
